# Spatial mapping of ectonucleotidase gene expression in the murine urinary bladder

**DOI:** 10.3389/fphys.2023.1306500

**Published:** 2023-11-30

**Authors:** Mafalda S. L. Aresta Branco, Brian A. Perrino, Violeta N. Mutafova-Yambolieva

**Affiliations:** Department of Physiology and Cell Biology, University of Nevada Reno School of Medicine, Reno, NV, United States

**Keywords:** ATP, bladder, nucleotidases, purinergic signaling, RNAscope

## Abstract

Purinergic signaling is important for normal bladder function, as it is thought to initiate the voiding reflex and modulate smooth muscle tone. The availability of adenine nucleotides and nucleosides (aka purines) at receptor sites of various cell types in the bladder wall is regulated by ectonucleotidases (ENTDs). ENTDs hydrolyze purines such as adenosine 5′-triphosphate (ATP) and adenosine 5′-diphosphate (ADP) with varying preference for the individual substrate. Therefore, the end effect of extracellular purines may depend significantly on the type of ENTD that is expressed in close proximity to the target cells. ENTDs likely have distinct cellular associations, but the specific locations of individual enzymes in the bladder wall are poorly understood. We used RNAscope™, an RNA *in situ* hybridization (ISH) technology, to visualize the distribution and measure the levels of gene expression of the main recognized ectonucleotidases in large high-resolution images of murine bladder sections. The relative gene expression of ENTDs was *Entpd3 > Alpl >> Enpp1 = Entpd2 >> Enpp3 > Entpd1* (very low to no signal) in the urothelium, *Entpd1 ≥ Entpd2 >> Enpp3 > Enpp1 = Alpl ≥ Nt5e* (very low to no signal) in the lamina propria, and *Entpd1 >> Nt5e = Entpd2 >> Enpp1 > Alpl = Enpp3* in the detrusor. These layer-specific differences might be important in compartmentalized regulation of purine availability and subsequent functions in the bladder wall and may explain reported asymmetries in purine availability in the bladder lumen and suburothelium/lamina propria spaces.

## Graphical Abstract

**Figure:**
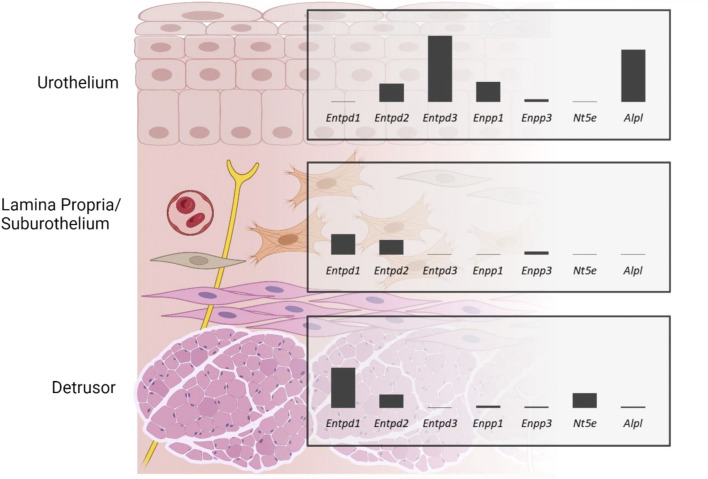
Gene expression of ectonucleotidases in the murine bladder wall demonstrates that ectonucleotidases are differentially expressed in detrusor, lamina propria, and urothelium of the murine bladder. Created with https://BioRender.com.

## 1 Introduction

Adenosine 5′-triphosphate (ATP) is released as a co-transmitter of acetylcholine by parasympathetic nerves and possibly as a co-transmitter of norepinephrine/noradrenaline by sympathetic nerves in the bladder (Burnstock, 2014) as well as by the urothelium into both luminal and suburothelial sides in response to bladder distension (Yu, 2015; Durnin et al., 2019). ATP released by efferent neurons can modulate smooth muscle tone via P2X_1_ receptors (Vial and Evans, 2000), whereas urothelial ATP can act in autocrine and paracrine ways via different P2X and P2Y receptor subtypes to stimulate urothelial cells (Ferguson et al., 2015; Chess-Williams et al., 2019), suburothelial interstitial cells/myofibroblasts (Wu et al., 2004; Cheng et al., 2011), and sensory nerves (Cockayne et al., 2000; Kaan et al., 2010). Of particular relevance, P2X_2/3_ and P2X_3_ receptor activation on sensory nerves is thought to convey the sensation of bladder fullness and initiate the micturition reflex in the bladder (Cockayne et al., 2000; Kaan et al., 2010).

Released ATP is rapidly hydrolyzed by membrane-bound and soluble forms of ectonucleotidases to ADP, AMP, and adenosine (ADO) (Yu, 2015; Aresta Branco et al., 2022; Gutierrez Cruz et al., 2022). It should be noted that ADP and ADO are biologically active metabolites that can modulate detrusor excitability via G-protein-coupled P2Y purinergic and A1, A2a, A2b, and A3 adenosine receptors, respectively. While ADP actions result in detrusor muscle contraction (Yu et al., 2014), ADO acts in an opposing way, evoking smooth muscle relaxation (Hao et al., 2019). Hence, extracellular metabolism of purines is a key determinant of the availability of these mediators at receptor sites and ensuing functions in the bladder.

Remarkably, the available concentration and relative proportion of purines are asymmetrical in the luminal and suburothelial sides of the urothelium at the end of bladder filling (Durnin et al., 2019), suggesting cell-specific differences in the release and/or metabolism of purines. Immunohistochemical studies report differences in the expression and distribution of ectonucleotidases in the bladder that may explain these observations (Yu et al., 2011; Yu, 2015; Babou Kammoe et al., 2021). Furthermore, in recent studies we observed distinct profiles of purine degradation in the lamina propria/suburothelium and intraluminal space, which, interestingly, was accompanied by the differential release of soluble ectonucleotidases into these compartments (Aresta Branco et al., 2022; Gutierrez Cruz et al., 2022). Previous immunohistochemical studies provided valuable information regarding the expression and distribution of some ectonucleotidase families in the bladder (Yu et al., 2011; Yu, 2015; Babou Kammoe et al., 2021). Nevertheless, a more comprehensive study was warranted. Therefore, we used RNAscope™—a more specific, sensitive, and reliable method—to assess the distribution and to measure the expression levels of several ectonucleotidase genes *(Entpd1, Entpd2, Entpd3, Enpp1, Enpp3, Nt5e and Alpl)* in the layers of the bladder (urothelium, lamina propria, and detrusor) and subset populations of the urothelium (basal plus intermediate cells and umbrella cells) across large high-resolution images of sagittal bladder sections. The results of this study provide strong support to the idea that ectonucleotidases are differentially expressed in the layers of the bladder wall likely providing specialized regulation of amount/type of excitatory and inhibitory purine mediators at effector cells.

## 2 Materials and methods

### 2.1 Animals

Male C57BL6J (3 months old, *n* = 3) were purchased from Jackson Laboratory (JAX stock #000664, Bar Harbor, MN). The mice were housed with free access to food and water and maintained in a 12 h light-dark cycle. The mice were sedated with isoflurane (AErrane; Baxter, Deerfield, IL, United States) and euthanized by cervical dislocation and exsanguination. All experimental procedures were carried out with the approval of the Institutional Animal Use and Care Committee at the University of Nevada, Reno and in accordance with the standards of the National Institutes of Health’s *Guide for the Care and Use of Laboratory Animals*.

### 2.2 Tissue preparation

Urinary bladders together with the proximal urethras and distal ureters were excised immediately after cervical dislocation and placed in oxygenated ice-cold Krebs-bicarbonate solution (KBS; mM: 118.5 NaCl, 4.2 KCl, 1.2 MgCl_2_, 23.8 NaHCO_3_, 1.2 KH_2_PO_4_, 11.0 dextrose, and 1.8 CaCl_2_; pH 7.4). After excess fat was removed, the whole bladders with connected proximal urethras and ureters were fixed in paraformaldehyde (PFA) 4% in phosphate-buffered saline (PBS) at 4°C for 24 h. The PFA solution was also added to the lumen of the bladder through the urethra to ensure adequate fixation. After several washes with PBS, the bladders and urethras were cut in half through the median plane with sharp blade and washed again in PBS three times at 30 min per wash. The preparations were cryopreserved by immersing the tissue for 15 min in increasing concentrations of sucrose in PBS [5%–20% (w/v)] and left overnight in 30% (w/v) sucrose in PBS at 4°C. The tissue preparations were embedded in a 1:1 mixture of 30% sucrose in PBS and O.C.T (Fisher, TX, United States), positioned in a cryomold with the cut side facing down, and frozen at −80°C. The blocks were cut into 14 µm sagittal cross sections using a Leica CM3050 S cryostat and placed onto VWR^®^ Premium Superfrost^®^ Plus Microscope Slides. Sections were left to air dry for 60 min at −20°C and then stored with desiccants at −20°C. Tissue preparation was carried out according to protocol provided by Advanced Cell Diagnostics (ACD, Bio-Techne, Newark, CA, United States) for fixed frozen tissue.

### 2.3 RNA *in situ* hybridization

Tissue preparations, pre-treatments, and RNAscope™ assays were performed according to ACD user manual (Document UM 323100) instructions for fixed frozen tissue, using RNAscope™ Multiplex Fluorescent Reagent Kit V2 (ACDBio Cat. No. 323100). Briefly, this involves sequential permeabilization of the tissue, hybridization of probes with target RNA, and amplification of signal. RNAscope™ probes were used to target *Krt20* (Cat. No. 402301-C3), *Krt5* (Cat. No. 415041 or 415041-C2), *Entpd1* (Cat. No. 475761), *Entpd2* (Cat. No. 579591-C2), *Entpd3* (Cat. No. 1236741-C1), *Enpp1* (Cat. No. 441191), *Enpp3* (Cat. No. 1236751-C1), *Nt5e* (Cat. No. 437951) and *Alpl* (Cat. No. 441161). As recommended by the manufacturer, the 3-plex Positive Control Probes [POLR2A (Channel C1), PPIB (Channel C2), UBC (Channel C3), Cat.No.320881] were used to help qualify samples and interpret assay results and the 3-plex Negative Control Probe (Cat. No. 320871) that targets the bacterial *dapB* gene was used to control for background noise and to help interpret assay results. The following Opal fluorophore reagents (Akoya Biosciences, MA, United States) were used to detect up to three targets in a single image: Opal™ 520 (Cat. No. FP1487001KT), Opal™ 570 (Cat. No. FP1488001KT), and Opal™ 690 (Cat. No. FP1497001KT). Nuclear counterstain was achieved with DAPI (included in the RNAscope™ Multiplex Fluorescent Reagent Kit V2). Slides were mounted with VECTASHIELD Vibrance^®^ Antifade Mounting Medium (Vector laboratories, CA, United States).

### 2.4 Image acquisition

Tissue sections were imaged with a Leica Stellaris 5 HyD S Confocal Microscope with a HC PL APO 40x/1,30 OIL CS2 lens (Leica, Wetzlar, Germany), using the laser lines 405 nm, 488 nm, 561 nm, and 638 nm and the following parameters: format 1024 × 1024, speed 600 Hz, zoom factor of 1, pinhole size 1.00 AU. Multi-tile images of the whole area of the sections combined with z-stacks (size 0.50 µm, 6-9 steps) were obtained.

### 2.5 Image analysis

**FIGURE 1:**
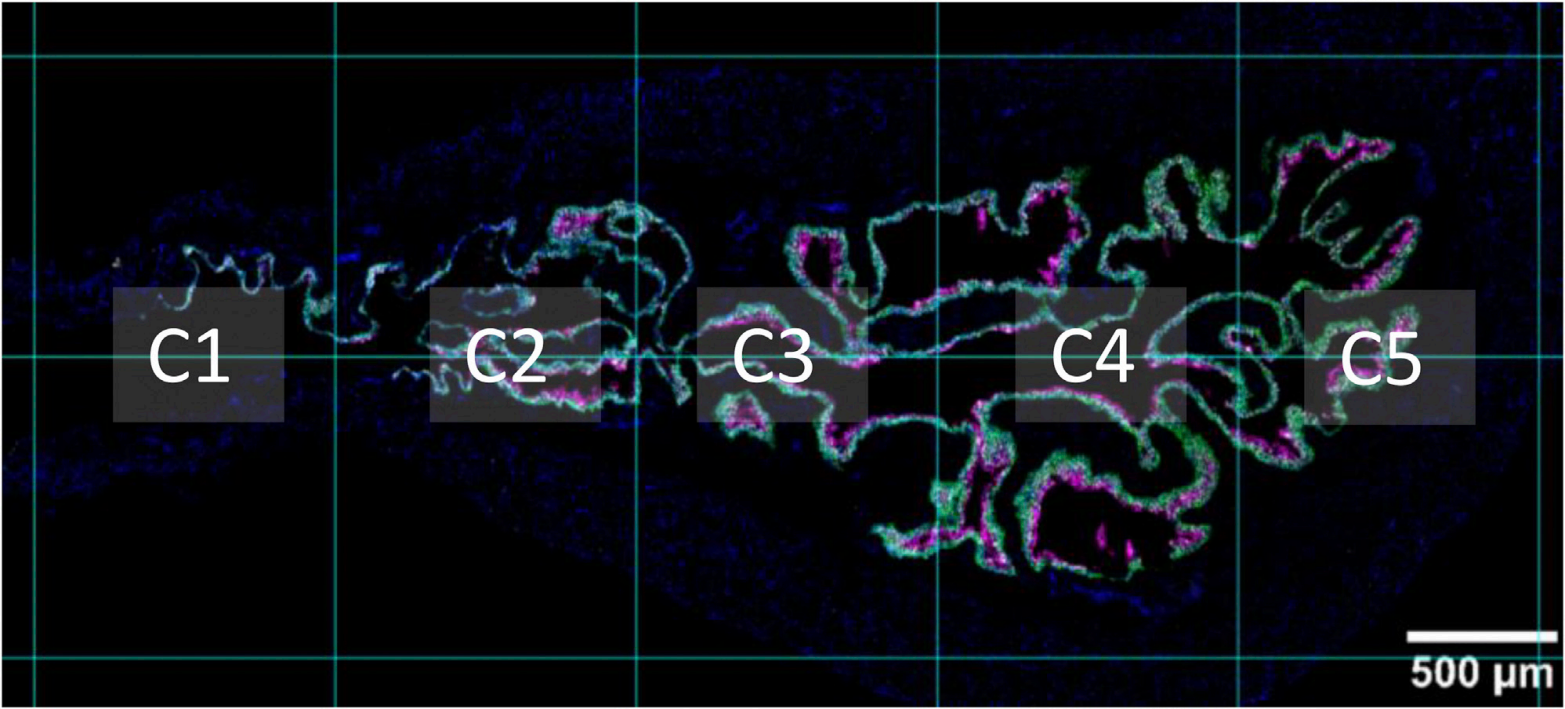
Sagittal view of proximal urethra and bladder. A supporting grid was used throughout analysis, which segmented the proximal urethra and bladder into five columns. Column one (C1) refers to the proximal urethra/neck region. Column two (C2) is the region that contains the trigone. Columns three to five include the bladder body. The apex of the bladder (bladder dome) can be seen in column five. Nuclei are shown in blue, *Krt5* expression is shown in green, and *Krt20* expression is shown in magenta.

Images were processed and analyzed using Fiji (Fiji is just ImageJ) software (Schindelin et al., 2012). Brightness and contrast were adjusted uniformly across all images. False colors were applied to each channel to optimize visual contrast and ensure the figures were color-blind safe. Nuclei are shown in blue and each nucleotidases is shown in the grey scale. *Krt5* expression (shown in green) was used to identify basal and intermediate urothelial cells and *Krt20* expression (shown in magenta) was used to identify umbrella cells, as these are well-established markers for these cell types (Colopy et al., 2014; Papafotiou et al., 2016), and were not subjected to further analysis. A grid that segmented the sagittal sections of proximal urethra and bladder in five columns was used to support analysis (Figure 1). The leftmost column (column one) refers to the proximal urethra/bladder neck region, column two contains the trigone region, whereas columns three to five include the body of the bladder (with the apex/dome situated in column five). This approach was chosen in order to identify possible regional differences/gradients from bladder neck to dome as demonstrated previously for bladder innervations (Pirker et al., 2005; Smith-Anttila et al., 2021). Two regions of interest of the detrusor (50 000 μm^2^), lamina propria (25 000 μm^2^), and urothelium (along a 250 µm straight line) per upper and bottom part of each column were selected for analysis (making for a total of 20 ROI per layer, per section). Each region of interest was only analyzed in one XY-plane. ACD guidelines for analysis of RNAscope data using Fiji/ImageJ were followed. Briefly, for each region of interest, the number of nuclei were counted by duplicating the original image, selecting the individual channel for DAPI, applying an appropriate threshold, and making the image binary, followed by analysis of the particles (size 4-infinite µm^2^, circularity 0-infinite). The detected particles were compared to the original image and the number of nuclei was corrected based on visual inspection, if necessary. In cases where segregation of nuclei was not successfully achieved, to allow for the use of “Analyze particles”, the multi-point tool was used to manually count each nucleus. Each dot referring to an ecto-nucleotidase RNA transcript was counted in a similar way, by applying the default 7–255 threshold with dark background, which we selected based on acquisition parameters and negative and positive controls, and making the image binary, followed by analysis of the particles (size 0.4-infinite µm^2^, circularity 0-infinite) in the region of interest. RNAscope signal is detected as punctate dots but clusters can result from overlapping signals from multiple mRNA molecules that are in close proximity to each other. The total probe count within each cluster was estimated by dividing the area of the cluster by a single probe area. Average number of dots/cell per layer was determined for each column. Results for columns 2–5 (excluding urethra) were averaged for each bladder section imaged (*n* = 3). Mean ± SD of dots/cell for each layer was plotted in graph bars. Additionally, results are reported as a score in accordance with the semi-quantitative scoring guideline provided by Advanced Cell Diagnostics (accessed at https://acdbio.com/dataanalysisguide) as follows: score 0: no staining or <1 dot/10 cells; score 1: 1-3 dots/cell; score 2: 4-9 dots/cell, no or very few dot clusters; score 3: 10–15 dots/cell and/or <10% of dots are in clusters; score 4: >15 dots/cell and/or >10% of dots are in clusters. Additionally, a score of 0.5 was used when the average number of dots was above 1 dot/10 cells but less than 0.5 dot/cell. The terms very low, low, medium, high, and very high expression used throughout the text refer to the scores of 0.5, 1, 2, 3, and 4, respectively.

## 3 Results

### 3.1 RNAscope™ sample validation

**FIGURE 2:**
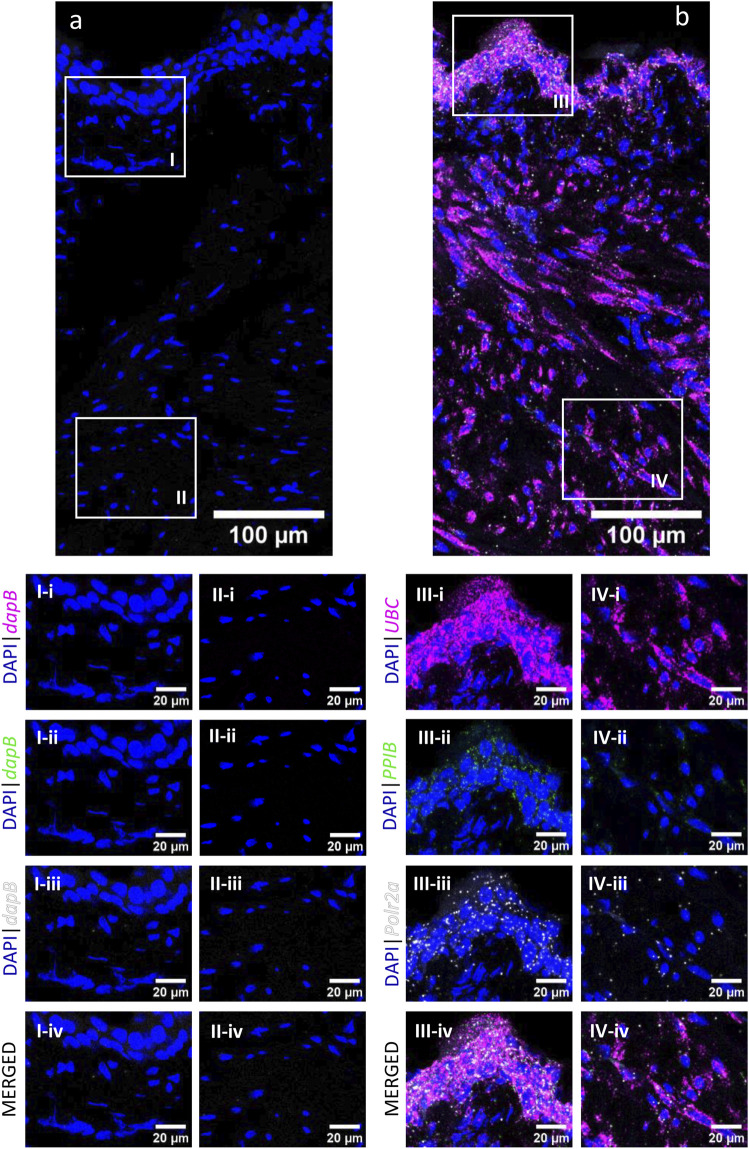
RNAscope 3-plex negative and positive controls in the murine urinary bladder. RNAscope 3-plex negative control, which targets the bacterial *dapB,* is shown in a representative cross-section of the bladder **(A)** and enlarged images of the mucosa **(I)** and detrusor **(II)**. RNAscope 3-plex positive control probes, which target murine *UBC* (magenta), *PPIB* (green) and *Polr2a* (grey) are depicted in a representative cross-section of the bladder **(B)** and enlarged images of the mucosa **(III)** and detrusor **(IV)**. Nuclear counterstaining with DAPI (blue) was applied.

The RNAscope 3-plex negative control (Figures 2a, I, II), which targets the bacterial *dabB* gene (dihydrodipicolinate *B. subtilis* reductase) generated a score of 0, thus confirming the absence of background noise in the study conditions. The RNAscope 3-plex positive control (Figures 2b, III, IV), which targets the mouse house-keeping genes *Polr2A* (RNA polymerase II subunit A), *PPIB* (peptidylprolyl isomerase B), and *UBC* (Ubiquitin C) generated scores of 1-2 (low to medium expression), 2–3 (medium to high expression), and 4 (very high expression), respectively. These results confirmed the integrity of the tissue.

### 3.2 Entpd1

**FIGURE 3:**
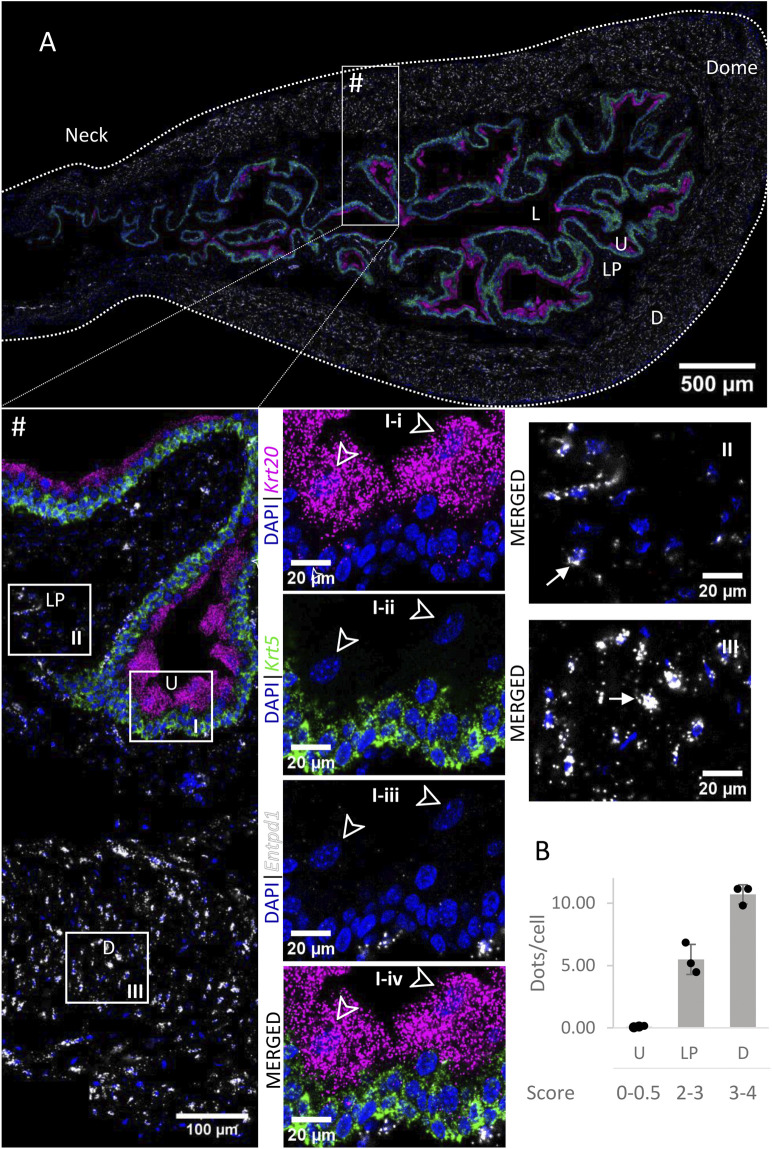
Spatial detection of *Entpd1* (grey scale), *Krt5* (green) and *Krt20* (magenta) mRNA transcripts in a cross-section of murine urinary bladder. Nuclear counterstaining with DAPI (blue) was applied. A high resolution large tiled image of the bladder cross-section **(A)** was obtained with a 4000× total magnification. L, bladder lumen; U, urothelium; LP, lamina propria; D, bladder detrusor. Enlarged images of all layers (#, flipped vertically), urothelium (U, **I**), lamina propria (LP, **II**) and detrusor muscle (D, **III**). *Krt5* mRNA detection was used to identify basal and intermediate urothelial cells, whereas very high detection of *Krt20* was used to identify umbrella cells (arrow heads, **I-i-iv**). *Entpd1* was predominantly detected in the lamina propria and detrusor (white arrows denote examples of *Entpd1* transcripts). Average dots/cell and score per layer **(B)** are shown for *Entpd1*.

High to very high *Entpd1* mRNA expression (Figure 3) was detected in the detrusor of the bladder. *Entpd1* expression was medium to high in the lamina propria of the bladder. However, the expression was low in the neck and proximal urethra region for both detrusor and lamina propria. *Entpd1* was mostly absent in the urothelium, although it was observed in a very small population of basal cells, often in clusters.

### 3.3 Entpd2

**FIGURE 4:**
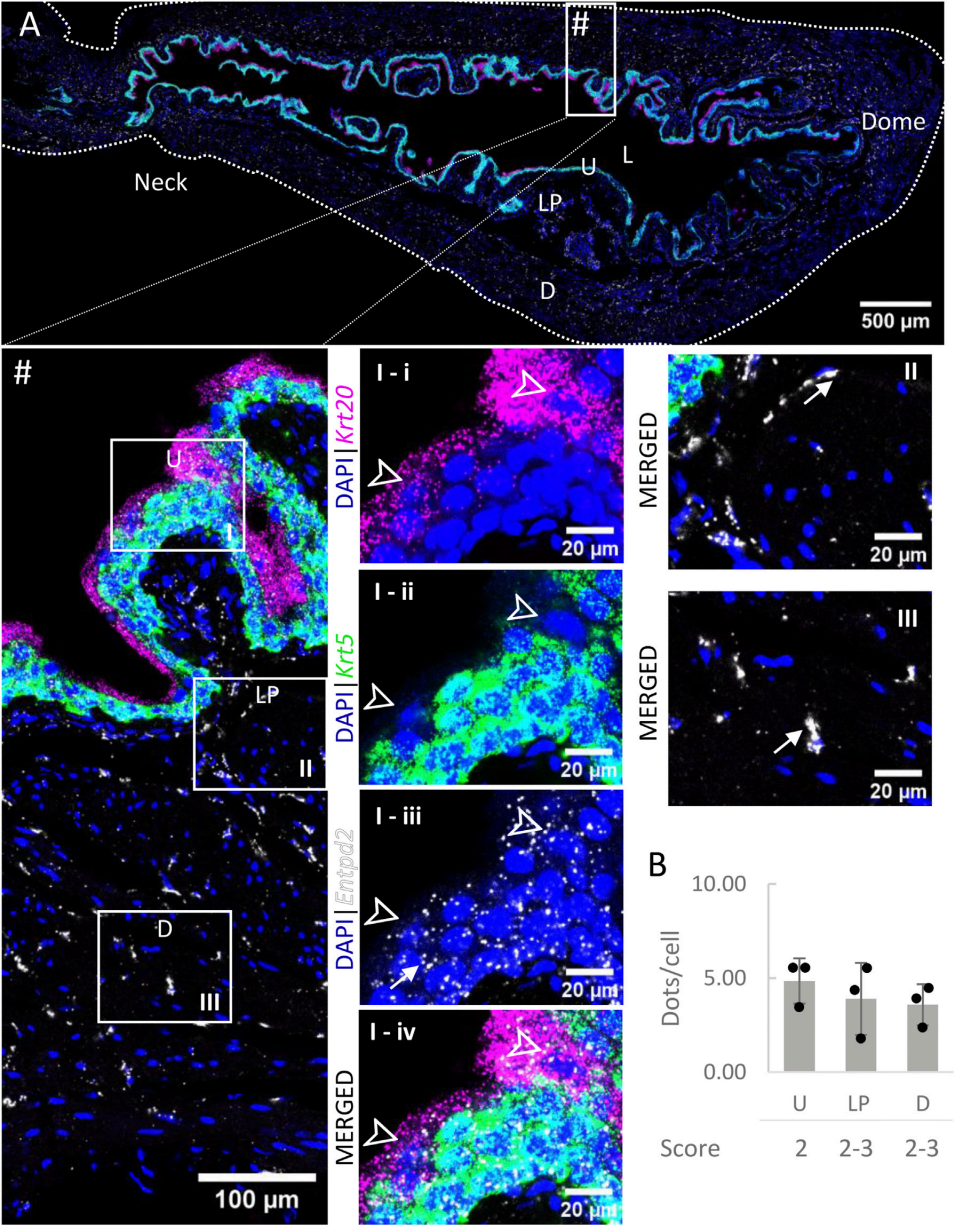
Spatial detection of *Entpd2* (grey scale), *Krt5* (green) and *Krt20* (magenta) mRNA transcripts in a cross-section of murine urinary bladder. Nuclear counterstaining with DAPI (blue) was applied. A high resolution large tiled image of the bladder cross-section **(A)** was obtained with a 4000× total magnification. L, bladder lumen; U, urothelium; LP, lamina propria; D, bladder detrusor. Enlarged images of all layers (#, flipped vertically), urothelium (U, **I**), lamina propria (LP, **II**) and detrusor muscle (D, **III**). *Krt5* mRNA detection was used to identify basal and intermediate urothelial cells, whereas very high detection of *Krt20* was used to identify umbrella cells (arrow heads, **I-i-iv**). *Entpd2* was detected in all layers, with medium expression. Average dots/cell and score per layer **(B)** are shown for *Entpd2*.

*Entpd2* mRNA transcripts (Figure 4) were found in all layers of the bladder, with similar levels of expression (∼4 dots/cell on average). As an exception, umbrella cells exhibited a very high expression of *Entpd2* (21.60 ± 5.29 dots/cell). The density of distribution of *Entpd2* was not remarkably different from the neck to dome of the bladder.

### 3.4 Entpd3

**FIGURE 5:**
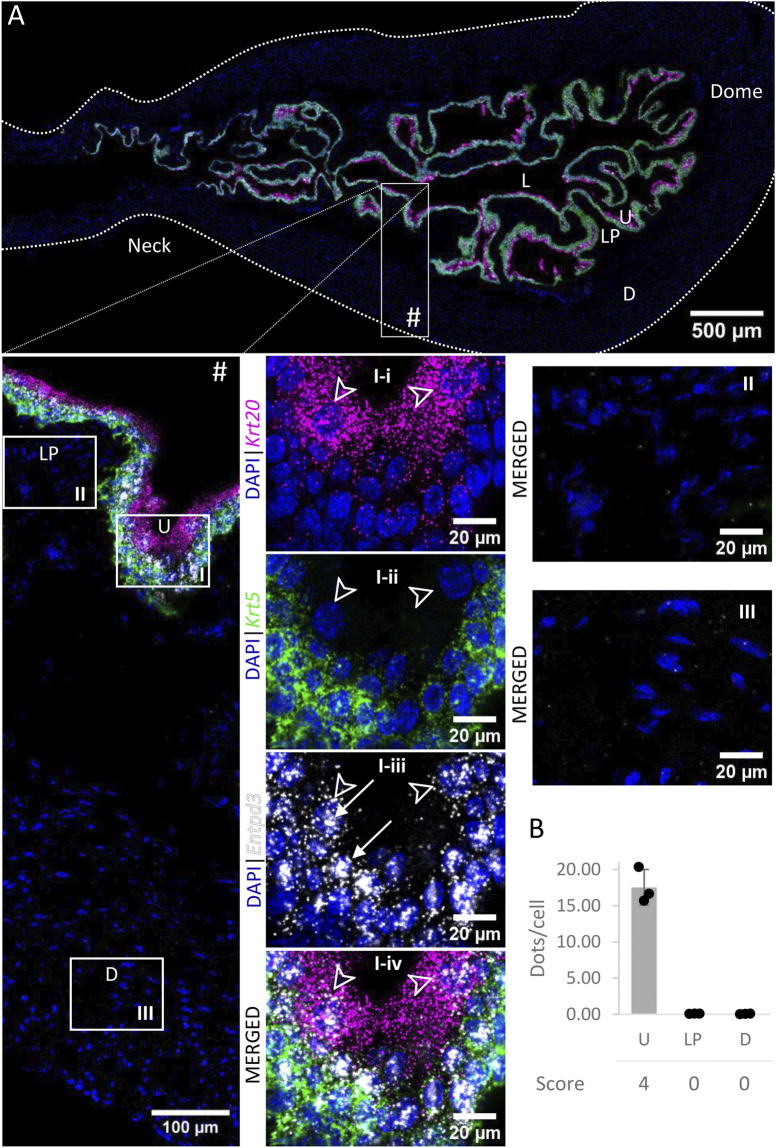
Spatial detection of *Entpd3* (grey scale), *Krt5* (green) and *Krt20* (magenta) mRNA transcripts in a cross-section of murine urinary bladder. Nuclear counterstaining with DAPI (blue) was applied. A high resolution large tiled image of the bladder cross-section **(A)** was obtained with a 4000× total magnification. L, bladder lumen; U, urothelium; LP, lamina propria; D, bladder detrusor. Enlarged images of all layers (#), urothelium (U, **I**), lamina propria (LP, **II**) and detrusor muscle (D, **III**). *Krt5* mRNA detection was used to identify basal and intermediate urothelial cells, whereas high detection of *Krt20* was used to identify umbrella cells (arrow heads, **I-i-iv**). *Entpd3* mRNA was found to be very highly expressed in urothelial cells (white arrows denote examples of *Entpd3* transcripts) but was absent in the detrusor and lamina propria. Average dots/cell and score per layer **(B)** are shown for *Entpd3*.

*Entpd3 mRNA* (Figure 5) was very highly expressed in the urothelium of the bladder body and medium to highly expressed in the urothelium of neck/proximal urethra region. The expression in umbrella cells was on average 48.15 ± 7.90 dots/cell. As the cell boundaries between basal and intermediate cells are not well defined, we did not differentiate between the two populations of cells in our measurements. However, we observed a clear trend of higher distributions of *Entpd3* dots in intermediate cells than basal cells. *Entpd3* was not found to be expressed in the detrusor nor in the lamina propria.

### 3.5 *Enpp1*

**FIGURE 6:**
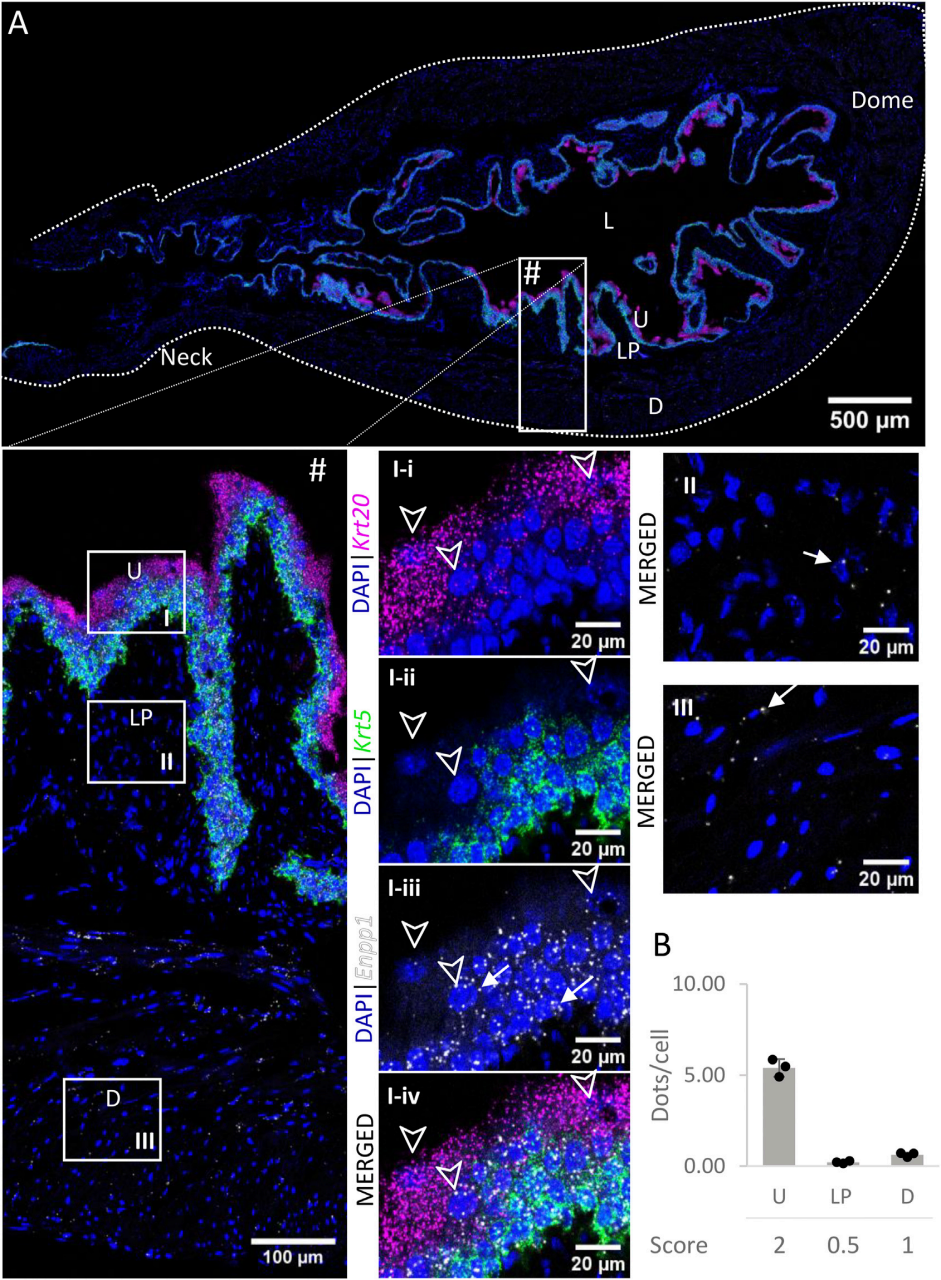
Spatial detection of *Enpp1* (grey scale), *Krt5* (green) and *Krt20* (magenta) mRNA transcripts in a cross-section of murine urinary bladder. Nuclear counterstaining with DAPI (blue) was applied. A high resolution large tiled image of the bladder cross-section **(A)** was obtained with a 4000× total magnification. L, bladder lumen; U, urothelium; LP, lamina propria; D, bladder detrusor. Enlarged images of all layers (#), urothelium (U, **I**), lamina propria (LP, **II**) and detrusor muscle (D, **III**). *Krt5* mRNA detection was used to identify basal and intermediate urothelial cells, whereas very high detection of *Krt20* was used to identify umbrella cells (arrow heads, **I-i-iv**). Urothelial cells exhibited a medium expression of *Enpp1* mRNA, whereas the lamina propria and detrusor showed a very low and low expression, respectively (white arrows denote examples of *Enpp1* transcripts). Average dots/cell and score per layer **(B)** are shown for *Enpp1*.

*Enpp1 mRNA* (Figure 6) was detected in the urothelium with medium expression. Levels of expression were similar across the layers of the urothelium. Expression was very low in the lamina propria and low in the detrusor. There was no significant difference between the distribution in the urothelium of the neck, body or dome of the bladder.

### 3.6 *Enpp3*

**FIGURE 7:**
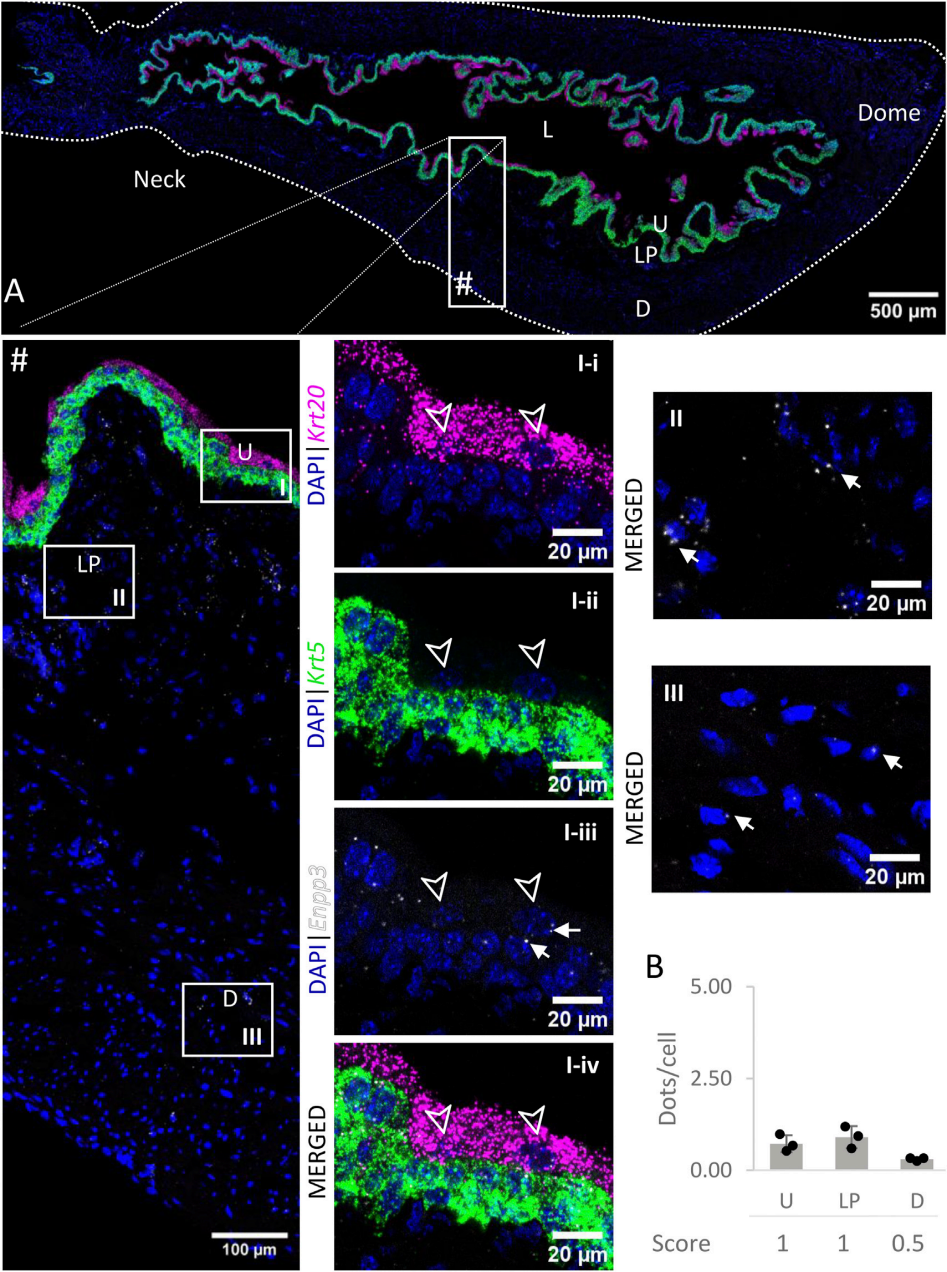
Spatial detection of *Enpp3* (grey scale), *Krt5* (green) and *Krt20* (magenta) mRNA transcripts in a cross-section of murine urinary bladder. Nuclear counterstaining with DAPI (blue) was applied. A high resolution large tiled image of the bladder cross-section **(A)** was obtained with a 4000× total magnification. L, bladder lumen; U, urothelium; LP, lamina propria; D, bladder detrusor. Enlarged images of all layers (#), urothelium (U, **I**), lamina propria (LP, **II**) and detrusor muscle (D, **III**). *Krt5* mRNA detection was used to identify basal and intermediate urothelial cells, whereas very high detection of *Krt20* was used to identify umbrella cells (arrow heads, **I-i-iv**). The urothelium and lamina propria exhibited a low expression of *Enpp3* mRNA, whereas the detection in the detrusor was very low (white arrows depict examples of *Enpp3* transcripts). Average dots/cell and score per layer **(B)** are shown for *Enpp3*.

The levels of expression of *Enpp3 mRNA* (Figure 7) were low in the urothelium and lamina propria, and very low in the detrusor across all regions.

### 3.7 *Nt5e*

**FIGURE 8:**
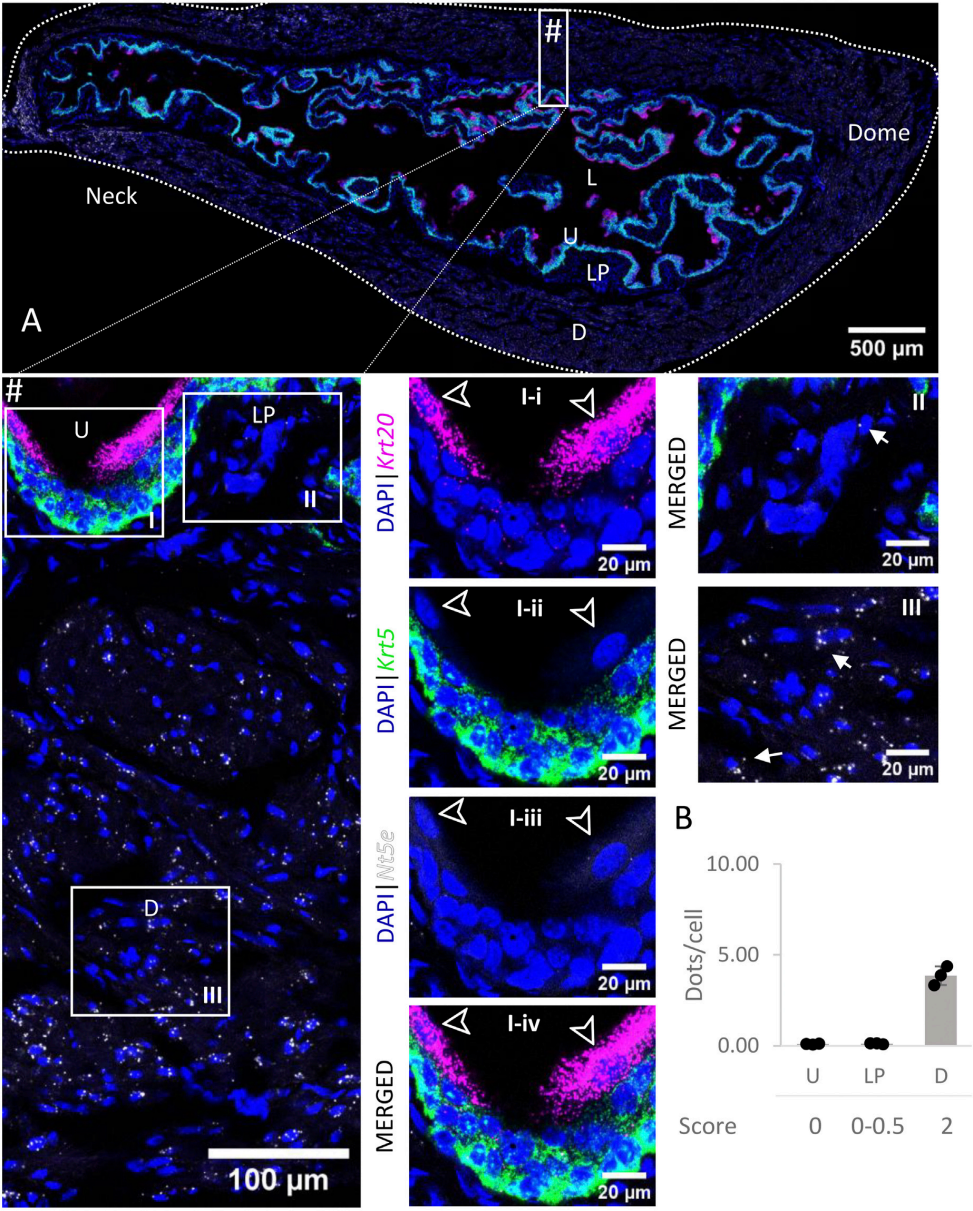
Spatial detection of *Nt5e* (grey scale), *Krt5* (green) and *Krt20* (magenta) mRNA transcripts in a cross-section of murine urinary bladder. Nuclear counterstaining with DAPI (blue) was applied. A high resolution large tiled image of the bladder cross-section **(A)** was obtained with a 4000× total magnification. L, bladder lumen; U, urothelium; LP, lamina propria; D, bladder detrusor. Enlarged images of all layers (#, flipped vertically), urothelium (U, **I**), lamina propria (LP, **II**) and detrusor muscle (D, **III**). *Krt5* mRNA detection was used to identify basal and intermediate urothelial cells, whereas very high detection of *Krt20* was used to identify umbrella cells (arrow heads, **I-i-iv**). *Nt5e* transcripts were mostly detected in the detrusor, with a medium level of expression. Average dots/cell and score per layer **(B)** are shown for *Nt5e*.

*Nt5e* mRNA transcripts (Figure 8) were detected in the detrusor, with medium levels of expression whereas the urothelium and lamina propria exhibited very low to no expression of *Nt5e*. In each layer, the levels of expression were similar across all regions of the bladder.

### 3.8 *Alpl*

**FIGURE 9:**
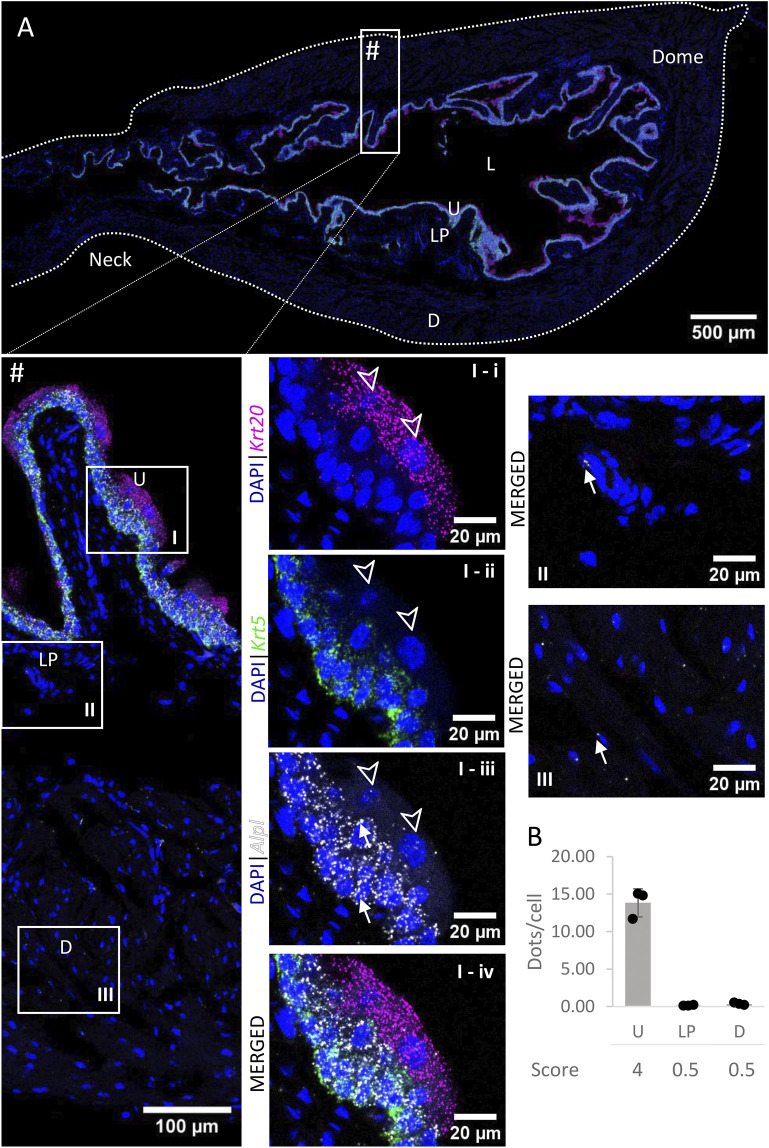
Spatial detection of *Alpl* (grey scale), *Krt5* (green) and *Krt20* (magenta) mRNA transcripts in a cross-section of murine urinary bladder. Nuclear counterstaining with DAPI (blue) was applied. A high resolution large tiled image of the bladder cross-section **(A)** was obtained with a 4000× total magnification. L, bladder lumen; U, urothelium; LP, lamina propria; D, bladder detrusor. Enlarged images of all layers (#, flipped vertically), urothelium (U, **I**), lamina propria (LP, **II**) and detrusor muscle (D, **III**). *Krt5* mRNA detection was used to identify basal and intermediate urothelial cells, whereas very high detection of *Krt20* was used to identify umbrella cells (arrow heads, I-i-iv). *Alpl* was predominantly detected in the urothelium (white arrows depict examples of *Alpl* transcripts). Average dots/cell and score per layer **(B)** are shown for *Alpl*.

Very high levels of *Alpl* mRNA (Figure 9) were detected in the basal and intermediate urothelial cells, whereas umbrella cells exhibited a medium expression (5.53 ± 2.09 dots/cell) of this gene. In contrast, expression levels were very low in the lamina propria and detrusor. There were no remarkable differences in levels of expression across the bladder regions.

## 4 Discussion

There are four main families of ectonucleotidases with different substrate affinity and specificity, namely ecto-nucleoside triphosphate diphosphohydrolases (ENTPDases; EC 3.6.1.5), ecto-nucleotide pyrophosphatase/phosphodiesterases (ENPPs; EC 3.6.1.9; EC 3.1.4.1), alkaline phosphatase/tissue-nonspecific isozyme (ALPL/TNAP; EC 3.1.3.1), and 5′-nucleotidase (NT5E/CD73; EC 3.1.3.5) (Zimmermann et al., 2012). ENTPDs differ significantly in product formation. This is of significant consequence for the regulation of nucleotide signaling. Thus, ENTPDs generally hydrolyze nucleoside triphosphate (e.g., ATP, UTP) and diphosphate (e.g., ADP, UDP), to generate nucleoside monophosphates as the final product (e.g., AMP, UMP). Among ENTPD1, ENTPD2, and ENTPD3, ENTPD1 has the highest affinity for ATP. ATP is hydrolyzed by ENTPD1 directly to AMP without significant amounts of ADP appearing as an intermediate (Heine et al., 1999; Kukulski et al., 2005). ENTPD2 and ENTPD3, on the other hand, hydrolyze ATP to ADP, which is released from the enzyme and further hydrolyzed to AMP. In the case of ENTPD2, considerable amounts of ADP accumulate before it is further hydrolyzed to AMP. ENPPs display broader substrate specificities, being able to hydrolyze nucleoside triphosphates and diphosphates as well as dinucleoside polyphosphates, ADP ribose, and nicotinamide adenine dinucleotide (NAD^+^), and a variety of artificial substrates, but not AMP. Of ENPP1 and ENPP2, ENPP1 hydrolyzes ATP to a higher degree than ENPP3 (Zimmermann et al., 2012). NT5E is nucleotide-specific and is regarded as the major enzyme that dephosphorylates AMP to generate extracellular ADO (Zimmermann, 2020). ALPL metabolizes a broad spectrum of substrates including 5′-nucleotides (ATP, ADP, and AMP), monophosphates, and pyrophosphate (Zimmermann et al., 2012). Therefore, ALPL is capable of catabolizing completely ATP to ADO providing an alternative pathway to NT5E for production of ADO. In general, all ENTPDs are highly active at physiological pH; they also differ in the breadth of optimal pH (Zimmermann et al., 2012). Since substrate preferences and product formation differ for many ENTDs, tissue distribution and cellular localization of individual ENTDs may determine the response of target cells to extracellular purines.

Functions of ENTDs can result in achieving effective agonist concentrations at receptor sites, prevention of receptor desensitization, termination of receptor activation or receptor activation by biologically active metabolites (Zimmermann et al., 2012). Numerous purinergic receptors are expressed throughout the bladder wall and regulate bladder excitability (Burnstock, 2014). However, tissue distribution and cellular localization of ENTDs in the bladder wall is not well understood. We focused our study on the distribution and gene expression of *Entpd1, Entpd2, Entpd3, Enpp1, Enpp3, Nt5e*, and *Alpl*, as these are thought to be the most prevalent ectonucleotidases in the bladder, with recognized protein cell-surface expression and functions in this organ (Yu et al., 2011; Babou Kammoe et al., 2021; Aresta Branco et al., 2022; Gutierrez Cruz et al., 2022). We used RNAscope, a commercially available RNA *in situ* hybridization (ISH) technology that is highly specific, sensitive, fast, reproducible, and a solid alternative or complement to immunohistochemistry techniques (Erben and Buonanno, 2019). This assay allows for multiplex detection for up to four target genes at a single cell level, within the spatial and morphological tissue context. Briefly, RNAscope uses oligonucleotide RNA probes with a Z design, consisting of bases complementary to the target-RNA which are linked to a preamplifier binding region. The probes are hybridized in pairs to form a landing platform for the preamplifier which then binds to an array of identical amplifiers, providing multiple binding sites for label probes, thus greatly enhancing the signal-to-noise ratio (Wang et al., 2012). The number of dots quantitatively represent mRNA levels and these dots can be compared between different probes (Erben and Buonanno, 2019; Jolly et al., 2019; Caldwell et al., 2021).

We found that 1) the genes of all seven ectonucleotidases tested are expressed in the murine bladder wall; 2) the relative expression of individual ectonucleotidases differs between the principal layers of the bladder wall (i.e., detrusor, lamina propria, and urothelium); 3) there were no clear regional differences in the mRNA expression of ectonucleotidases with the exception of the neck region in which *Entpd1* and *Entpd3* showed lower expression in the detrusor and urothelium, respectively.

In this study, we show that *Entpd1* is the most expressed ectonucleotidase gene in the detrusor and lamina propria, with high to very high and medium to high mRNA expressions in the bladder body, respectively. These results together with an absence of signal in the urothelium are consistent with the distribution of ENTPD1 protein reported in two immunohistochemical studies in murine bladders (Yu et al., 2011; Babou Kammoe et al., 2021). ENTPD1 was also found to be the most expressed ectonucleotidase in the detrusor using RT-qPCR (Babou Kammoe et al., 2021) and in mucosa homogenates using automated capillary based immunodetection Wes technology (Aresta Branco et al., 2022). Furthermore, ENTPD1 was found in releasable/soluble form in concentrated extraluminal (i.e., from the lamina propria side) solutions (cELS) collected from distended detrusor-free bladder preparations (Aresta Branco et al., 2022), but not in intraluminal solutions (ILS) using the same model (Gutierrez Cruz et al., 2022). In the presence of membrane-bound ENTPD1, ATP is hydrolyzed almost directly to AMP (Robson et al., 2006). Therefore, ENTPD1 should terminate the activation of ADP-specific (e.g., P2Y1,12,13) receptors far more efficiently than the other ENTPDs. Functional studies have shown more potent muscle contractions evoked by nucleotides in detrusor strips from *Entpd1* ^−/−^ mice than wild-type mice (Babou Kammoe et al., 2021). Together, these findings suggest a critical role of ENTPD1 in terminating the actions of ATP (and ADP) in the lamina propria and detrusor layers. Interestingly, we observed that *Entpd1* expression was lower towards the bladder neck and proximal urethra, which might be an important regional difference in the regulation of smooth muscle tone. Regional differences have been described for the innervation of the bladder (Fowler et al., 2008). For example, a prominent suburothelial plexus of sensory nerves is described in the bladder base and neck, whereas such nerves are relatively sparse at the bladder dome (Gabella and Davis, 1998). Parasympathetic nerves that mediate contraction during micturition are dominant in the detrusor of the bladder body but sympathetic nerves that contribute to continence are widespread in the bladder neck, but sparsely distributed in the muscle (Beckel and Holstege, 2011).Very little is known about regional differences in purinergic signaling in the bladder. Experiments in pigs and mini-pigs have suggested that purinergic innervation may play a role at the start of micturition by activation of P2X receptors and inducing the initial detrusor muscle contraction and at the same time relaxing the bladder neck via P2Y receptor stimulation to facilitate micturition (Hernández et al., 2009; Burnstock, 2014). It is possible that lower *Entpd1* expression is associated with higher preservation of ATP concentrations at P2Y purinergic receptors that mediate relaxation of the bladder neck and proximal urethra during micturition.

Here, we report that *Entpd2* mRNA transcripts are present in all layers, including urothelium, with identical levels of expression. In immunohistochemical studies it was suggested that ENTPD2 proteins are localized between smooth muscle bundles and in the lamina propria, but not in the urothelium (Yu et al., 2011; Babou Kammoe et al., 2021). However, ENTPD2 was found in low concentration in ILS of detrusor-free bladder preparations, which supports protein expression in the urothelium and regulated release into the lumen (Gutierrez Cruz et al., 2022). ENTPD2 was also found expressed in mucosa homogenates and cELS of detrusor-free bladder preparations using Wes (Aresta Branco et al., 2022). RT-qPCR performed in smooth muscle cells of the detrusor also confirmed *Entpd2* expression in this layer (Babou Kammoe et al., 2021). Additionally, pharmacological studies showed a stronger inhibition of ATP degradation in cELS and cILS by POM-1 (a polyoxometalate that inhibits ENTPD1, 2 and 3 (Müller et al., 2006)) more than ARL67156 (a competitive inhibitor of ENTPD1, ENTPD3, and ENPP1 (Lévesque et al., 2007)), which can in part be explained by differences in specificity towards ENTPD2 (Aresta Branco et al., 2022). ENTPD2 is expected to promote the activation of ADP specific receptors, because in the presence of ATP it produces a sustained accumulation of ADP (Kukulski et al., 2005; Zimmermann et al., 2012).

*Entpd3* mRNA expression was restricted to the urothelium and was the most prevalent in this layer. Signal appeared to be greater in intermediate and umbrella cells than in basal urothelial cells. *Entpd3* expression in the urothelium was slightly lower in the bladder neck and proximal urethra than in the bladder body, which might be relevant in specialized regional regulation of purines. ENTPD3 immunolocalization has also been reported to be limited to the urothelium (Yu et al., 2011; Yu, 2015; Babou Kammoe et al., 2021), although signal distribution across the urothelial layers seems to differ slightly depending on the antibody used. In agreement with our results, ENTPD3 was the major soluble ectonucleotidase released into the bladder lumen of detrusor-free preparations at the end of bladder filling (Gutierrez Cruz et al., 2022). ENTPD3 was also detected using Wes in bladder mucosa preparations and was the second largest soluble ectonucleotidase released into the lamina propria space (Aresta Branco et al., 2022), which might have originated from regulated release by the basal urothelial layer. ENTPD3 shows substrate preference of ATP over ADP, and causes moderate accumulation of ADP in the presence of ATP. Therefore it is likely to lead to transient ADP accumulation and activation of ADP-specific receptors (Kukulski et al., 2005). Overall, these findings suggest that ENTPD3 is the major ATPase and ADPase produced in the urothelium, which not only contributes to urothelial hydrolysis of ATP to AMP but is also likely aiding the regulation of purines availability and subsequent activity in the lamina propria as a result of urothelial enzyme release.

ENPPs exhibit nucleotide pyrophosphatase and phosphodiesterase enzymatic activity to generate nucleoside 5′-monophosphates (Borza et al., 2022). To our knowledge the distribution of ENPP1 and ENPP3 in the bladder has not been described previously. In this study we report that *Enpp1* transcripts are mostly expressed in the urothelium, with similar levels of expression as *Entpd2*. In agreement with this result, we have previously shown that ENPP1 is the second most prevalent enzyme in the pool of soluble ectonucleotidases released into the bladder lumen (Gutierrez Cruz et al., 2022). ENPP1 has also been detected by PCR in porcine bladder (Petersen et al., 2007) and by Wes in the mucosa of murine bladder (Aresta Branco et al., 2022). ENPP1 displays a high catalytic efficiency in hydrolyzing ATP to AMP with formation of inorganic pyrophosphate (Borza et al., 2022). Therefore, ENPP1 is likely to play a role in hydrolysis of ATP to AMP in the urothelium and bladder lumen, without accumulation of ADP.

*In situ* hybridization also revealed low to very low levels of expression of *Enpp3* in the bladder. We previously reported low levels of ENPP3 protein expression in the murine bladder mucosa (Aresta Branco et al., 2022), as well as a low release of this enzyme into the lamina propria and luminal spaces. ENPP3 shows low specificity for the different nucleotides, presenting only a two-fold preference for ATP (Borza et al., 2022). It is possible that ENPP3 has only a minor role in bladder purinergic signaling.

*Nt5e* transcripts were found in the detrusor layer primarily. This is in agreement with immunohistochemical findings (Yu et al., 2011; Babou Kammoe et al., 2021) and RT-qPCR results showing that *Nt5e* was the second most expressed ectonucleotidase in the detrusor (Babou Kammoe et al., 2021). Therefore, NT5E is likely the major AMPase in the detrusor. *Nt5e* mRNA expression was difficult to resolve in the urothelium or LP in the present study. However, low levels of NT5E in the mucosa of the bladder, cELS and cILS of detrusor-free bladder preparations were detected previously using Wes methodology (Aresta Branco et al., 2022; Gutierrez Cruz et al., 2022). Remarkably, degradation of 1,*N* ^6^-etheno-AMP (eAMP, a highly fluorescent analog of AMP) to eADO was significantly hampered in ILS of *Nt5e* ^*−/−*^ preparations, when compared to wild-type (Aresta Branco et al., 2022). This suggests that even at low levels of expression, the catalytic activity of NT5E may be a determining factor of the AMP/ADO ratio in the bladder mucosa.

Expression of *Alpl* mRNA was higher in basal and intermediate urothelial cell than in umbrella cells, which was in agreement with immunohistochemical observations (Yu, 2015). However, this is in discrepancy with our Wes data that showed low expression of this enzyme in the bladder mucosa (Aresta Branco et al., 2022). It is possible that the antibody for ALPL/TNAP was not of sufficient quality to detect the enzyme accurately. ALPL/TNAP shows wide substrate specificity, thus it is able to sequentially hydrolyze ATP all the way to ADO (Zimmermann et al., 2012). Differential distribution of ALPL in the urothelium can help explain asymmetries in purine metabolism in the basal and apical layers of the urothelium.

**FIGURE 10:**
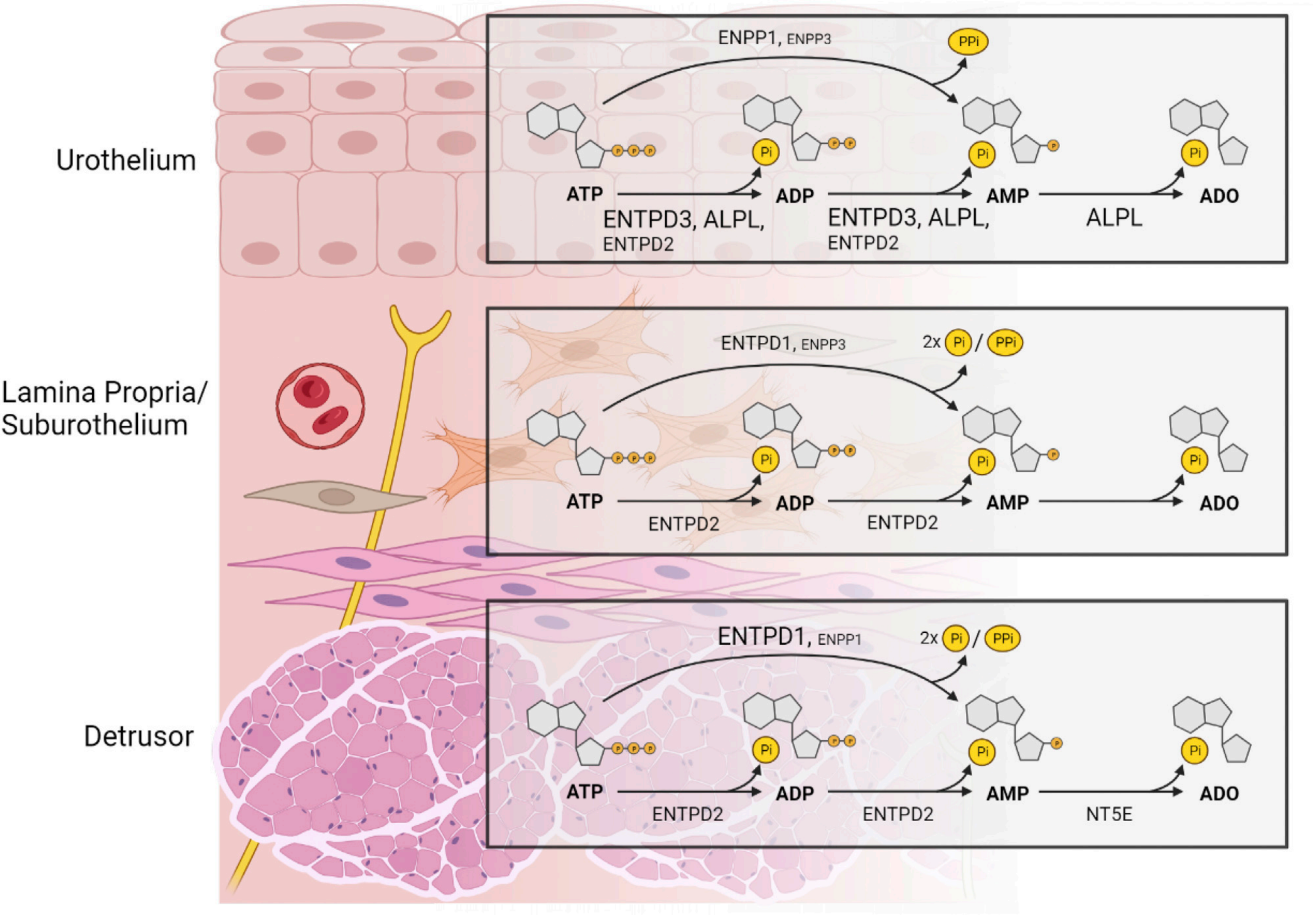
A model depicting the distribution of ectonucleotidases responsible for product formation with substrate ATP in key layers of the murine bladder wall that is based on the distribution of mRNA of selected ectonucleotidases. The size of the label indicates the relative expression of an ectonucleotidase. In the urothelium, ENTPD3 and ALPL and to a lesser degree ENTPD2 dephosphorylate sequentially ATP to ADP and AMP. ATP is also directly hydrolyzed to AMP and inorganic pyrophosphate (PPi) by ENPP1, and to a lesser degree by ENPP3 whereas production of ADO from AMP is exclusively mediated by ALPL. ENTPD1 and NT5E do not have a substantial role in adenine nucleotide and nucleoside formation in the urothelium. In the lamina propria/suburothelium, ENTPD1 catalyzes ATP to AMP and two phosphate groups (2xPi), with minimal accumulation of ADP, whereas ENTPD2 hydrolyzes sequentially ATP to ADP and AMP with sustained accumulation of ADP. AMP in the lamina propria can also, to a lesser extent, be formed directly from ATP by ENPP3. Lamina propria/suburothelium lacks enzymatic machinery to produce adenosine from extracellular adenine nucleotides. Therefore, presence of ADO in the suburothelium/lamina propria is likely a result of ADO release through nucleoside transporters. In addition, ADO might result from activity of soluble ectonucleotidases released into this space from adjacent layers. In the detrusor layer, ADP and AMP are produced by ENTPD2-mediated hydrolysis of ATP, whereas AMP is the main product of ENTPD1 catalysis and to a lesser degree of ENPP1. NT5E is the primary enzyme responsible for the formation of ADO from AMP in the detrusor muscle layer. The activation of specific purinergic receptors at precise loci within the bladder wall depends on the hydrolysis of extracellular purines mediated by multiple ectonucleotidases with various substrate specificity and product formation. The regulation of adenine nucleotides (purine) signaling in the bladder wall is remarkably complex. Created with https://BioRender.com.

In conclusion, this study highlights the differences in gene expression of the main ectonucleotidases in the murine bladder and provides a rationale for regional and layer-specific asymmetries in the metabolism of purines and resultant function. Based on our results, a compartmentalized regulation of extracellular purine concentrations in the layers of the bladder wall should be expected (Figure 10). This provides a solid foundational work for future studies aimed at understanding a possible role of ectonucleotidases in bladder dysfunction.

## Data Availability

The raw data supporting the conclusion of this article will be made available by the authors, without undue reservation.
